# One Coin, Two Sides: Eliciting Expert Knowledge From Training Participants in a Capacity-Building Program for Veterinary Professionals

**DOI:** 10.3389/fvets.2021.729159

**Published:** 2021-10-25

**Authors:** Julie Adamchick, María Sol Pérez Aguirreburualde, Andres M. Perez, Mary Katherine O'Brien

**Affiliations:** ^1^Department of Veterinary Population Medicine, College of Veterinary Medicine, University of Minnesota, Saint Paul, MN, United States; ^2^Center for Animal Health and Food Safety, College of Veterinary Medicine, University of Minnesota, Saint Paul, MN, United States

**Keywords:** expert elicitation, capacity building, veterinary medicine, research, education, Kenya, Uganda

## Abstract

Scientific research may include the elicitation of judgment from non-academic subject-matter experts in order to improve the quality and/or impact of research studies. Elicitation of expert knowledge or judgment is used when data are missing, incomplete, or not representative for the specific setting and processes being studied. Rigorous methods are crucial to ensure robust study results, and yet the quality of the elicitation can be affected by a number of practical constraints, including the understanding that subject-matter experts have of the elicitation process itself. In this paper, we present a case of expert elicitation embedded within an extended training course for veterinary professionals as an example of overcoming these constraints. The coupling of the two activities enabled extended opportunities for training and a relationship of mutual respect to be the foundation for the elicitation process. In addition, the participatory research activities reinforced knowledge synthesis objectives of the educational program. Finally, the synergy between the two concurrent objectives may produce benefits which transcend either independent activity: solutions and ideas built by local professionals, evolving collaborative research and training approaches, and a network of diverse academic and practicing professionals. This approach has the versatility to be adapted to many training and research opportunities.

## Introduction

Scientific research may include non-academic participants in the research process to improve the quality and impact of studies (1–3). There are many paradigms, methodologies, and purposes for utilizing such approaches. This paper focuses on the elicitation of knowledge from subject matter experts, whose estimation or judgment of fact-based matters is used to answer the research question (3, 4). This approach is utilized when available data are scarce, unrepresentative, or inadequate to describe the processes and systems being studied. “Expert” in this usage refers to a person who can provide information about the question based on their experience with the subject matter of interest (5, 6).

Expert elicitation is increasingly common within veterinary science, although used less frequently than in other fields. A search on Web of Science for “expert knowledge” OR “expert elicitation” OR “expert judgment” returned 60 articles (out of 708,779) within the category of Veterinary Sciences, 30 of which were published since 2017. When accounting for the total number of articles in each Web of Science category, the same search string occurred 10 times more frequently within Environmental Sciences (1,232/1,489,989) and 12 times more frequently for Ecology (599/591,636). The purposes of expert knowledge in veterinary publications include estimation of parameter values (7, 8), ranking of risk factors or criteria (9–11), enhancing or interpreting available data (12–14), or developing an instrument for use by practitioners (15, 16). Many applications are in data-scarce environments, but there are also cases where expertise is used to make sense of or add rigor to abundant or heterogeneous data sources (14, 17).

When expert knowledge is utilized as a source of information, there are limitations and potential pitfalls (18). People have restricted mental models, poor causal reasoning, and are prone to a litany of biases (4, 19). Estimating probabilities and quantifying uncertainty require training distinct from subject matter expertise (4). Rigorous and structured procedures for participant selection, knowledge elicitation and interpretation, and study validation are crucial to ensure the quality of study conclusions (3, 4, 20).

Structured procedures and training of participants can help to alleviate bias but may be inconvenient or impractical, especially when working with subject matter experts from outside of academia. Elicitations may be carried out in a restricted time period (e.g., embedded within a workshop or conference) or through long-distance interactions. Including participants who are “boots on the ground” practitioners or community members can be challenging if they have limited time available for the activity and a steeper learning curve with respect to the research and elicitation methods. Subject matter experts may not have an academic understanding of the techniques being used, which can impede effective communication and impact the quality of the results if adequate training is not provided.

In this paper, we present a case of expert elicitation embedded within an extended training course for veterinary professionals as an example of overcoming some of these constraints. The coupling of the two activities may create a synergy between research and training which enriches the outcomes and expands the impact of each component, creating a whole greater than the sum of the parts. First, we give a brief overview of the training program, research objectives, and expert elicitation activities performed. Then, we describe the observed outcomes and character of this approach, perceived to be beneficial and synergistic. Finally, we discuss considerations for future opportunities.

## Research and Training Overview

The research objective was to quantify and analyze the risk for transmission of foot and mouth disease (FMD) associated with the export of beef produced in Kenyan and Ugandan cattle systems. FMD is a highly infectious transboundary disease of cattle and other livestock and wildlife species (21) and is endemic to East African countries (22, 23). In order to model that risk, it was necessary to understand the underlying processes and the values of key variables. Most of those data are not published; people who work in those beef cattle systems provided expertise and guidance to build, quantify, and validate the risk assessment model.

The elicitation was carried out within 2 concurrent cohorts of ProgRESSVet: a systematic education program for building professional capacity of veterinarians in Kenya and Uganda delivered by the University of Minnesota Center for Animal Health and Food Safety (CAHFS) (24). Participants for the program in each country were required to have a degree in veterinary medicine and experience in the field. There were 13 veterinarians from Kenya, with an average of 13 (range of 2–29) years of experience working in animal health and/or production. The Ugandan cohort had 10 participants, with an average of 7 years of experience (range 2–15 years).

ProgRESSVet training programs are tailored to address gaps identified in the OIE (World Organization for Animal Health) Performance of Veterinary Services Pathway (25) for each country or region of implementation. The programs are designed per Fink (26) to build individual capacity to generate lasting change in participants, thereby building the technical, collaborative, and systems-thinking capacity of the Veterinary Services (VS) to ultimately improve the health and well-being of the communities and countries where they work (24). ProgRESSVet was first offered in 2017 and in 2018 in the Latin American region. ProgRESSVet Uganda and ProgRESSVet Kenya were launched in 2020, incorporating new educational elements based on results of formative and summative education evaluation from the previous Latin America program.

**Figure 1:**
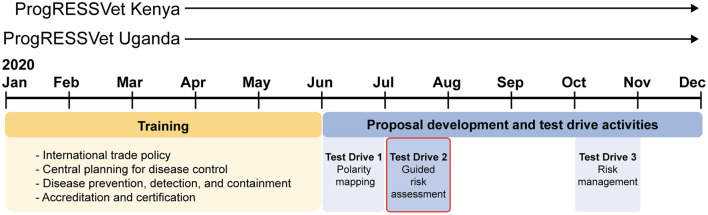
Research and training activities were carried out within 2 concurrent cohorts of ProgRESSVet: one in Kenya and one in Uganda. Participants completed 5 months of online coursework followed by 6 months developing proposals to support the trade of animals and animal products. The guided risk assessment was one of three “Test Drive” activities integrated into the curriculum which included participants in the process of data collection and synthesis about questions relevant to their own communities. The Test Drives, including the guided risk assessment, were part of the training program; participants could opt in for their contributions to be used for research purposes. All training and research activities were carried out separately in each country.

The guided risk assessment and elicitation was one of three activities integrated into the curriculum, which we called Test Drives (Figure 1). The Test Drives included participants in the process of data collection and synthesis about questions relevant to their own communities without requiring them to autonomously direct their own analyses. These activities were conceptualized to achieve research objectives during the challenges of covid-19 restrictions and were then recognized as an opportunity to support knowledge application.

Prior to the Test Drives, including the guided risk assessment, participants had completed 5 months of online coursework (Figure 1), including modules on risk analysis applied to animal health, food safety, and international trade. For the next 6 months, participants would develop proposals to support the trade of animals and animal products. Each portion of the training was structured and delivered by the same team of researchers and faculty. The guided risk assessment was part of the training program; participants could opt in for their contributions to be used for research purposes and 100% of enrolled individuals in each country chose to do so.

The details of the elicitation procedures and results are described elsewhere (27). The approach followed a modified version of the Delphi method, a technique for obtaining the consensus of a group of experts (28), and was carried out independently with the participants from Uganda and from Kenya (*n* = 10 and *n* = 13, respectively). First, participants individually worked through a series of open-ended questions in which they described the system, identified important variables and relationships, and critiqued a preliminary scenario tree and risk model structure. Next, also individually, they estimated the distributions for key parameter values. Both questionnaires are included as Supplementary Materials 1, 2. Those responses were synthesized and presented in a group discussion with each cohort in order to reach consensus on the meaning and values of key variables. Each participant received a final report with an accessible summary of the discussion and had the opportunity to comment with any additional suggestions or concerns.

## Research Process and Outcomes

The novelty of this approach was the use of an education program to support the elicitation activity and research objectives. Structured protocols recommend training experts in the elicitation approach and rationale being used (3, 29). Such training is thought to reduce apprehension, increase understanding of the process, provide motivation, identify biases among the experts, and provide guidelines for working between the facilitators and experts (4). However, practical constraints may preclude the incorporation of training into the research activities.

By embedding the elicitation within an extended educational program, several of these objectives were achieved. After 6 months of partnership (including adaptations on both sides to continue the program through covid-19 uncertainty), the experts (veterinary participants) and researchers (education team) had a collaborative working relationship with established norms and patterns. The researchers supported the participants in developing proposal ideas, which may have helped to convey the team's interest and investment in the individual and institutional impact to result from the program. The participants in each country knew one another through interactive ProgRESSVet activities, including pre-covid in-person workshops and a program discussion thread on the WhatsApp platform.

The education program also provided subject matter training for the exercise. The participants discussed the importance of the problem (the control challenges and trade repercussions of endemic FMD) throughout the courses. The curriculum included 5 weeks on risk analysis including probability and scenario trees, and the elicitation activities included supplemental training on these topics. The participants were well-versed in both “the how” and “the why” of the research question.

The ongoing engagement (in contrast to a single day or workshop) enabled an iterative process of elicitation, consultation, and consensus. Participants allocated a suggested 6–10 h per week to the program and were offered continuous professional development credit. This may have increased their motivation and time available to submit thorough and thoughtful responses. And the platform of a training program supported inclusion of expert participants who were on-the-ground practitioners across a variety of regions and roles in Kenyan and Ugandan livestock systems.

The attributes of the data collected—elicited, analyzed, and evaluated separately for Kenya and Uganda—reflects the value of this approach. Responses provided extensive descriptions of cattle health, production, and handling relevant to the research question. Candid discussions reflected participant perspectives of how the animal health system does work, not merely how it should work, including contrasts between distinct settings (e.g., feedlot vs. pastoralist). They provided insights about causal relationships based on firsthand experience, including the actions, motivations, and incentives of key actors. Participants took the option of responding “no answer” to some questions and/or focusing on specific production systems, suggesting to the researchers that they did not feel pressured to provide information beyond the extent of their experience.

As a result, valuable parameters were quantified by expert knowledge where there otherwise were no available data, and participant expertise improved the structure and specification of the risk model used to represent the system (17). Participants contributed information that otherwise may have been neglected and corrected errors in the researcher's thinking. For example, they highlighted the need to specify both disease diagnosis and appropriate follow-up action to define infected cattle as detected. They described scenarios in which the sale of cattle for meat may be correlated with the probability of having disease, and consequently an additional set of parameters was included to represent disease prevalence among animals which had been sold (rather than assuming animals chosen for sale would be selected at random). Both of these issues were raised by multiple individuals in each country.

## Synergistic Character

This coupled approach of training and expert elicitation yielded benefits beyond the research results. We would characterize the elicitation in this context as synergistic learning (26), complementing and enhancing the educational material rather than “stealing time” away from training. The Test Drives are intended to contribute to ProgRESSVet learning objectives by enabling participants to apply the tools presented to their own work, to have an expanded view of food systems and their roles, and to value the critical use of evidence for decision-making.

Participant responses to the end-of-program evaluation (supplied anonymously) support the perceived value of the Test Drive activities in contributing to these objectives. Several respondents said they had already applied the principles and skills from the Test Drives, including for work related to covid-19, animal disease control strategies, enhanced safety of meat, managing animal health challenges with limited resources, and even for embarking on a family project. Others commented on changes in their perspectives, including how to consider stakeholders affected by an issue, new understanding of regional and international trade, the multidimensional nature of livestock health challenges, finding common ground among partners with diverse perspectives, and sharing knowledge with other members of a One Health district task force. [The program evaluation asked about the suite of three Test Drive activities as a whole, so these responses describe skills and perspective garnered from the guided risk assessment as well as two other applied activities whose outputs were not used for research (Figure 1)].

**Figure 2:**
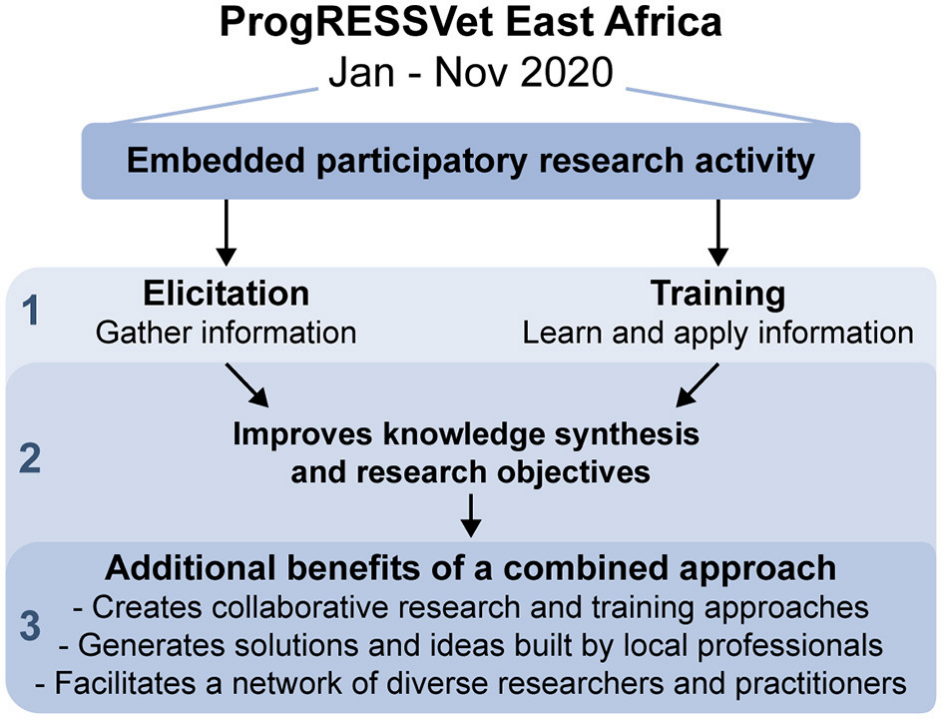
Benefits of combined elicitation and training activity embedded within an education program. *Level 1:* Both objectives (elicitation and education) can be achieved within a single activity. *Level 2:* Each attribute (elicitation and education) of the activity enhances the other, contributing to improved achievement of each. For example, the coupling of the two activities enabled extended opportunities for subject-matter training and a relationship of mutual respect to be the foundation for the elicitation process. The participatory research activities reinforced knowledge synthesis objectives of the educational program. *Level 3:* The synergy between the two concurrent objectives may produce benefits which transcend either independent activity: solutions and ideas built by local professionals, evolving collaborative research and training approaches, and a network of diverse academic and practicing professionals.

We believe the impact of this approach can transcend that of elicitation or training activities alone to produce benefits for the research and training team, the participants and their community, and the network of both (Figure 2). The experience and insights have contributed to the evolving culture of practice and specifically the education and training model at CAHFS: reinforcing and clarifying the ProgRESSVet approach as a collaborative engagement with peers from a diverse set of background experiences, cultures, and knowledge, focused on meeting local needs through building local capacity. The hope and intention is that participants were empowered by generating and synthesizing shared knowledge about the problems and processes studied, building individual and institutional capacity to address specific and unknown future challenges. Finally, the engagement helped create a network of professionals from both the university and Veterinary Services who can continue to work and learn together.

Future offerings of the ProgRESSVet curriculum will maintain the Test Drive approach and the education team will continue reporting related educational modifications and outputs pursuant to a robust understanding of the method's potential.

## Discussion

The coupling of research activities with capacity-building of health professionals has been applied previously (30, 31), though we have not seen a model in which the same individuals occupy the role of both trainees and contributors of expert knowledge. The ProgRESSVet and Test Drive approach is unique in that expert elicitation activities are embedded and structurally scaf folded within a broader training program, serving to complement the capacity-building objectives while eliciting and activating the expertise of the participants.

We believe this is a valuable approach with flexibility to adapt to particular settings and constraints. However, it is important to be aware of limitations or potential pitfalls. For example, in our case the experts were all veterinarians and nearly all employed in the public sector. A wider diversity of value chain actors would have provided more perspectives contributing to the research and to the discussion of local issues among participants (5). Our structured elicitation and consensus process was heavily facilitated; a constructivist approach with a more open-ended, participant-driven dialogue would favor a different paradigm of research themes and shared learning (2, 32).

The design and implementation of a similar program will require evaluation of the components (the participants, training, and research or elicitation activities) and how they fit together. Practitioners should weigh the value and tradeoffs of possible program designs, considering available resources, existing infrastructure, and their highest priority objectives. The research requiring participant input needs to be carefully aligned with participant expertise and experience. The type and scope of participatory research activity should be guided by the educational approach in order to complement other training elements. The research activity must be realistic given the duration of the training program and the relationships that will be established before launching the elicitation exercises. Time and effort required (of the participants and of the academic team) should be considered, including sequential or iterative steps for the research process.

As with any research method, it is critical to use systematic and robust methods for expert elicitation in order to obtain results that can withstand “close interrogation” and “independent validation,” two facets of reproducibility (33, 34). Rigorous approaches emphasize the inclusion of multiple and diverse experts and the use of a structured protocol for the phases of knowledge elicitation, aggregation, and validation (6, 29); the specific character of those methods may be situation-specific (35, 36). There is much yet to be studied about the nature of expert elicitation approaches that alleviate bias to obtain accurate and well-calibrated results (4).

Research studies that embed expert elicitation into a training program as described here should be designed to produce rigorous results, and may have opportunities to validate those results through repetition over multiple training cohorts. In addition, it may be possible to assess the impact of the coupled approach on the quality of research outputs, furthering the field's understanding of the practice and methodology of expert elicitation (4). For example, the impact on quantitative parameter estimates could be studied in the future by eliciting the parameterization from each participant before and after the training program. Another area of research could be to assess the relationship between responses and certain features of the participants (e.g., gender, age, years of experience). It may be expected that the training approach results in less variation in the responses, compared to gathering data in the absence of a training program, and may be less biased by external factors.

We have demonstrated the opportunity to gather information from subject matter experts in a way that enhances the research process and outputs while at the same time educating and training participants. In our experience, combining both objectives in a single set of activities served to reinforce each component. The participants, before their formal role as “experts,” were trained in the methods and rationale of risk analysis and had developed a relationship of mutual respect with the academic team members. Conversely, the experience of switching roles and interacting (with the subject matter and with each other) in a new way provided an opportunity for significant learning for the participants, pushing them beyond consumption of information or hypothetical scenarios into a realm of application to their actual communities and challenges, while able to sit in the seat of expertise to “test drive” research and analytic methodologies without the full expectation of designing and managing a project on their own. This combined approach has the potential to generate benefits for the academic team as well as the participants and their communities that transcend what any individual activity or institution would produce alone.

## Data Availability Statement

The original contributions presented in the study are included in the article/Supplementary Material, further inquiries can be directed to the corresponding author/s.

## Ethics Statement

Ethical review and approval was not required for the study on human participants in accordance with the local legislation and institutional requirements. The patients/participants provided their written informed consent to participate in this study.

## Author Contributions

JA drafted the manuscript. JA, MP, AP, and MO'B contributed to manuscript revision, read, and approved the submitted version. All authors contributed to the development and implementation of the project described.

## Funding

This project has been supported in part by the Bill & Melinda Gates Foundation (Grant No. OPP1211169). Under the grant conditions of the Foundation, a Creative Commons Attribution 4.0 Generic License has already been assigned to the author accepted manuscript version that might arise from this submission. Additional support was received from the USDA National Needs (Grant No. 2014-38413-21825) and from the MnDRIVE Global Food Ventures Graduate Student Professional Development award.

## Conflict of Interest

The authors declare that the research was conducted in the absence of any commercial or financial relationships that could be construed as a potential conflict of interest.

## Publisher's Note

All claims expressed in this article are solely those of the authors and do not necessarily represent those of their affiliated organizations, or those of the publisher, the editors and the reviewers. Any product that may be evaluated in this article, or claim that may be made by its manufacturer, is not guaranteed or endorsed by the publisher.

## Supplementary Material

Supplementary Material 1 Short answer survey.

Supplementary Material 2 Quantitative survey.
