# Supplementary material for: One Coin, Two Sides: Eliciting Expert Knowledge From Training Participants in a Capacity-Building Program for Veterinary Professionals
Source: Front Vet Sci. 2021 Oct 25;8:729159. doi: 10.3389/fvets.2021.729159 (PMC8573137; doi:10.3389/fvets.2021.729159)
Supplement: Supplementary Material 2 — Quantitative survey. [file Data_Sheet_2.PDF]

ProgRESSVet Uganda: risk analysis values

Start of Block: Background

Q1.1
Welcome to the ProgRESSVet risk analysis questionnaire!

The questionnaire starts with a background section containing 4 questions. Here, you will choose which subpopulations (any/all) you would like to provide your opinion and insight about.

For each subpopulation, there are 4 sections (Cattle population and disease risk, Sale and transport, Antemortem inspection and detection, Postmortem inspection and processing) with 4-7 questions per question.

We ask that you dedicate enough time to this to provide high quality information where possible. The amount of time will vary by how many subpopulations you choose, but we anticipate that it will take approximately 2-4 hours to complete.

Responses to the survey are saved every time you move to the next page, so you may leave the survey and return later to finish. As long as you return to the survey on the same computer and same web browser, and have not cleared your browser cookies, the questionnaire will resume where you left off.

Q1.2 **Background**

Q1.3
Name
We ask for your name only to know whether or not your questionnaire has been submitted; we will not record or present the answers in a way that is connected with identifying information.

____

Q1.4 How many years have you been working in animal health and/or production?

____

Q1.5 How would you describe your experience with cattle and beef production and processing in Uganda?

○ Expert (many first-hand experiences, have relationships with a diverse set of value chain stakeholders) (1)

○ Moderate (familiar with production and processing systems, some first-hand experiences) (2)

○ Limited (little experience, most knowledge is second-hand) (3)

Q1.6
We are assessing the cattle industry as 4 sub-populations: Pastoral, Agro-Pastoral, Ranching, and Semi-intensive. The descriptions of each can be found in the Part A and Part B documents.

For which sub-populations do you have experience and/or feel comfortable giving your opinions about FMD risk and the farm-to-market process?
Please choose all that apply.

▢ Pastoral (1)

▢ Agro-Pastoral (2)

▢ Ranching (3)

▢ Semi-intensive (4)

Q1.7 Do you perceive there to be severe limitations to using this classification of sub-populations? Please explain.

○ Yes (1) ____

○ No (2) ____

End of Block: Background

Start of Block: Sub-pop block

Q2.1
Block A: Cattle population and FMD risk during production
${lm://Field/1} systems

Please answer the following questions considering ONLY cattle raised in ${lm://Field/1} production systems.

After each question, please use the Reasoning field to provide 1-2 sentences explaining why you chose those values.
If you do not feel comfortable answering any question, please respond "No Answer".

Q2.2 Please briefly describe your experience with ${lm://Field/1} cattle production systems.

____

____

____

____

____

Q2.3 (P1) How many cases of foot and mouth disease occur in cattle per year (in ${lm://Field/1} systems)?

◯ Maximum: # cases per year (1) ____

◯ Minimum (5) ____

◯ Most likely (2) ____

◯ Reasoning (6) ____

Q2.4
(P1) What is the total population of cattle (in ${lm://Field/1} systems)?

◯ Maximum: # cattle (1) ____

◯ Minimum (2) ____

◯ Most likely (3) ____

◯ Reasoning (4) ____

Q2.5 (n) What % of the cattle population is sold for meat each year?

◯ Maximum: % of cattle population (0-100) (1) ____

◯ Minimum (2) ____

◯ Most likely (3) ____

◯ Reasoning (4) ____

Q2.6 (n) How many head of cattle are sold to be slaughtered for meat each year?

- ◯ Maximum: # of cattle (1) ____
- ◯ Minimum (2) ____
- ◯ Most likely (3) ____
- ◯ Reasoning (4) ____

Q2.7 Do you have any additional comments about FMD risk in the cattle population of ${lm://Field/1} systems?

____

Page Break

Q2.8
Block B: FMD risk during sale and transportation
${lm://Field/1} systems

Please answer the following questions considering ONLY cattle raised in ${lm://Field/1} production systems.

After each question, please use the Reasoning field to provide 1-2 sentences explaining why you chose those values.
If you do not feel comfortable answering any question, please respond "No Answer".

Q2.9 (P7, P8) How much time (days) passes between when a cow leaves the herd and slaughter?

○ Maximum: days (1) ____

○ Minimum (2) ____

○ Most likely (3) ____

○ Reasoning (4) ____

Q2.10 (P2) Is it possible that a bovine sold for meat is commingled with animals from other herds after leaving its own herd?

○ Yes (1)

○ No (2)

Display This Question:

If Loop current: (P2) Is it possible that a bovine sold for meat is commingled with animals from other herds after... = Yes

Q2.11 (P2) Please list the species of animals with which cattle may be commingled after leaving their own herd.

____

Display This Question:

If Loop current: (P2) Is it possible that a bovine sold for meat is commingled with animals from other herds after... = Yes

Q2.12 (P2) What % of cattle sold for meat are commingled with animals from other herds after leaving their own farms?

○ Maximum: % of cattle sold (0-100) (1) ____

○ Minimum (2) ____

○ Most likely (3) ____

○ Reasoning (4) ____

Display This Question:

If Loop current: (P2) Is it possible that a bovine sold for meat is commingled with animals from other herds after... = Yes

Q2.13 (P2) When commingling does occur, how many "new" cattle (from other herds) is an animal mixed with?

○ Maximum: # cattle (1) ____

○ Minimum (2) ____

○ Most likely (3) ____

○ Reasoning (4) ____

Display This Question:

If Loop current: (P2) Is it possible that a bovine sold for meat is commingled with animals from other herds after... = Yes

Q2.14 (P9, P10) At what step in the process from herd to slaughter does commingling first occur?

____

Display This Question:

If Loop current: (P2) Is it possible that a bovine sold for meat is commingled with animals from other herds after... = Yes

Q2.15 (P9, P10) How much time (days) passes between initial commingling with animals from other herds and slaughter?

- ◯ Maximum: days (1) ____
- ◯ Minimum (2) ____
- ◯ Most likely (3) ____
- ◯ Reasoning (4) ____

Q2.16 Do you have any additional comments about FMD risk during sale and transportation of cattle from ${lm://Field/1} systems?

____

Page Break

Q2.17

Block C: Antemortem inspection and detection of FMD-infected cattle

${lm://Field/1} systems

Please answer the following questions considering ONLY cattle raised in ${lm://Field/1} production systems.

After each question, please use the Reasoning field to provide 1-2 sentences explaining why you chose those values.
If you do not feel comfortable answering any question, please respond "No Answer".

Q2.18 (P3, P4) Are cattle inspected for FMD between farm/herd and slaughter?

- ◯ Yes (1)
- ◯ No (2)

Display This Question:

If Loop current: (P3, P4) Are cattle inspected for FMD between farm/herd and slaughter? = Yes

Q2.19 (P3, P4) Is the FMD inspection process different for cattle that are commingled with other animals during the sale process than for cattle that are only exposed to animals from their own herd? Differences could include: number of inspections, proficiency of person conducting the inspection, sensitivity of the inspection to detect an infected animal, or other differences.

- ◯ Yes, inspections are different for animals that are commingled vs. for animals that are only exposed to animals from their own herd (1)
- ◯ No, inspections are the same for all animals (2)
- ◯ Not Applicable, all animals are commingled with others during the sale process (3)

Display This Question:

If (P3, P4) Is the FMD inspection process different for cattle that are commingled with other animal... = Yes, inspections are different for animals that are commingled vs. for animals that are only exposed to animals from their own herd

Q2.20 Please explain the differences in the inspection process.

____

____

____

____

____

Display This Question:

If Loop current: (P3, P4) Are cattle inspected for FMD between farm/herd and slaughter? = Yes

Q2.21 (P3, P4) How many times is an animal potentially inspected or tested for FMD between farm and slaughter?

- ○ 1 (1)
- ○ 2 (2)
- ○ 3 (3)
- ○ 4 or more (4)

Display This Question:

If Loop current: (P3, P4) Are cattle inspected for FMD between farm/herd and slaughter? = Yes

Q2.22 (P3, P4) Briefly describe the first point at which an animal may be inspected (by whom, step in the sale/transport process, outcome if found to be positive or negative for FMD, etc.)

____

____

____

____

____

Display This Question:

If Loop current: (P3, P4) Are cattle inspected for FMD between farm/herd and slaughter? = Yes

Q2.23 (P3, P4) What % of cattle sold for meat undergo this inspection?

○ Maximum: % of cattle sold (0-100) (1)
____

○ Minimum (2) ____

○ Most likely (3) ____

○ Reasoning (4) ____

Display This Question:

If Loop current: (P3, P4) Are cattle inspected for FMD between farm/herd and slaughter? = Yes

Q2.24
(P5, P6) What % of FMD-infected cattle displaying clinical signs are identified as FMD-positive by this inspection? (In other words, what is the sensitivity of this inspection to detect FMD in this population of cattle?)

○ Maximum: % of infected cattle detected (0-100) (1)
____

○ Minimum (2) ____

○ Most likely (3) ____

○ Reasoning (4) ____

Display This Question:

If Loop current: (P3, P4) Are cattle inspected for FMD between farm/herd and slaughter? = Yes

Q77 (not on Event Tree) What % of cattle identified as FMD-positive by this inspection are NOT removed from the food supply, but instead the inspection results are ignored or compromised?

- ○ Maximum: % of positive test results ignored or compromsed (1) ____
- ○ Minimum (2) ____
- ○ Most likely (3) ____
- ○ Reasoning (4) ____

Display This Question:

If Loop current: (P3, P4) How many times is an animal potentially inspected or tested for FMD between farm and sla... = 2

Or Loop current: (P3, P4) How many times is an animal potentially inspected or tested for FMD between farm and sla... = 3

Or Loop current: (P3, P4) How many times is an animal potentially inspected or tested for FMD between farm and sla... = 4 or more

Q2.25 (P3, P4) Briefly describe the second point at which an animal may be inspected (by whom, step in the sale/transport process, outcome if found to be positive or negative for FMD, etc.)

____

____

____

____

____

Display This Question:

If Loop current: (P3, P4) How many times is an animal potentially inspected or tested for FMD between farm and sla... = 2

Or Loop current: (P3, P4) How many times is an animal potentially inspected or tested for FMD between farm and sla... = 3

Or Loop current: (P3, P4) How many times is an animal potentially inspected or tested for FMD between farm and sla... = 4 or more

Q2.26 (P3, P4) What % of cattle sold for meat undergo this inspection?

◯ Maximum: % of cattle sold (0-100) (1) ____

◯ Minimum (2) ____

◯ Most likely (3) ____

◯ Reasoning (4) ____

Display This Question:

If Loop current: (P3, P4) How many times is an animal potentially inspected or tested for FMD between farm and sla... = 2

Or Loop current: (P3, P4) How many times is an animal potentially inspected or tested for FMD between farm and sla... = 3

Or Loop current: (P3, P4) How many times is an animal potentially inspected or tested for FMD between farm and sla... = 4 or more

Q2.27
(P5, P6) What % of FMD-infected cattle displaying clinical signs are identified as FMD-positive by this inspection? (In other words, what is the sensitivity of this inspection to detect FMD in this population of cattle?)

◯ Maximum: % of infected cattle detected (0-100) (1) ____

◯ Minimum (2) ____

◯ Most likely (3) ____

◯ Reasoning (4) ____

Display This Question:

If Loop current: (P3, P4) How many times is an animal potentially inspected or tested for FMD between farm and sla... = 2

Or Loop current: (P3, P4) How many times is an animal potentially inspected or tested for FMD between farm and sla... = 3

Or Loop current: (P3, P4) How many times is an animal potentially inspected or tested for FMD between farm and sla... = 4 or more

Q78 (not on Event Tree) What % of cattle identified as FMD-positive by this inspection are NOT removed from the food supply, but instead the inspection results are ignored or compromised?

- ◯ Maximum: % of positive test results ignored or compromsed (1) ________________
- ◯ Minimum (2) ________________
- ◯ Most likely (3) ________________
- ◯ Reasoning (4) ________________

Display This Question:

If Loop current: (P3, P4) How many times is an animal potentially inspected or tested for FMD between farm and sla... = 3

Or Loop current: (P3, P4) How many times is an animal potentially inspected or tested for FMD between farm and sla... = 4 or more

Q2.28 (P3, P4) Briefly describe the third point at which an animal may be inspected (by whom, step in the sale/transport process, outcome if found to be positive or negative for FMD, etc.)

____

____

____

____

____

Display This Question:

If Loop current: (P3, P4) How many times is an animal potentially inspected or tested for FMD between farm and sla... = 3

Or Loop current: (P3, P4) How many times is an animal potentially inspected or tested for FMD between farm and sla... = 4 or more

Q2.29 (P3, P4) What % of cattle sold for meat undergo this inspection?

○ Maximum: % of cattle sold (1) ____

○ Minimum (2) ____

○ Most likely (3) ____

○ Reasoning (4) ____

Display This Question:

If Loop current: (P3, P4) How many times is an animal potentially inspected or tested for FMD between farm and sla... = 3

Or Loop current: (P3, P4) How many times is an animal potentially inspected or tested for FMD between farm and sla... = 4 or more

Q2.30
(P5, P6) What % of FMD-infected cattle displaying clinical signs are identified as FMD-positive by this inspection? (In other words, what is the sensitivity of this inspection to detect FMD in this population of cattle?)

○ Maximum: % of infected cattle detected (0-100) (1) ____

○ Minimum (2) ____

○ Most likely (3) ____

○ Reasoning (4) ____

Display This Question:

If Loop current: (P3, P4) How many times is an animal potentially inspected or tested for FMD between farm and sla... = 3

Or Loop current: (P3, P4) How many times is an animal potentially inspected or tested for FMD between farm and sla... = 4 or more

Q79 (not on Event Tree) What % of cattle identified as FMD-positive by this inspection are NOT removed from the food supply, but instead the inspection results are ignored or compromised?

○ Maximum: % of positive test results ignored or compromsed (1) ____

○ Minimum (2) ____

○ Most likely (3) ____

○ Reasoning (4) ____

Display This Question:

If Loop current: (P3, P4) How many times is an animal potentially inspected or tested for FMD between farm and sla... = 4 or more

Q2.31 (P3, P4) Briefly describe the fourth point at which an animal may be inspected (by whom, step in the sale/transport process, outcome if found to be positive or negative for FMD, etc.)

____

____

____

____

____

Display This Question:

If Loop current: (P3, P4) How many times is an animal potentially inspected or tested for FMD between farm and sla... = 4 or more

Q2.32 (P3, P4) What % of cattle sold for meat undergo this inspection?

◯ Maximum: % of cattle sold (1) ____

◯ Minimum (2) ____

◯ Most likely (3) ____

◯ Reasoning (4) ____

Display This Question:

If Loop current: (P3, P4) How many times is an animal potentially inspected or tested for FMD between farm and sla... = 4 or more

Q2.33

(P5, P6) What % of FMD-infected cattle displaying clinical signs are identified as FMD-positive by this inspection? (In other words, what is the sensitivity of this inspection to detect FMD in this population of cattle?)

◯ Maximum: % of infected cattle detected (0-100) (1) ____

◯ Minimum (2) ____

◯ Most likely (3) ____

◯ Reasoning (4) ____

Display This Question:

If Loop current: (P3, P4) How many times is an animal potentially inspected or tested for FMD between farm and sla... = 4 or more

Q80 (not on Event Tree) What % of cattle identified as FMD-positive by this inspection are NOT removed from the food supply, but instead the inspection results are ignored or compromised?

- ◯ Maximum: % of positive test results ignored or compromsed (1) ____
- ◯ Minimum (2) ____
- ◯ Most likely (3) ____
- ◯ Reasoning (4) ____

Display This Question:

If Loop current: (P3, P4) How many times is an animal potentially inspected or tested for FMD between farm and sla... = 4 or more

Q2.34 Are there additional inspections between sale and slaughter that you have not yet described?

- ◯ Yes (1)
- ◯ No (2)

Display This Question:

If Are there additional inspections between sale and slaughter that you have not yet described? = Yes

Q2.35
For each additional inspection point, please use the space below to describe:

- brief description of the inspection
- % of cattle sold for meat that undergo this inspection (max, min, most likely)
- % of infected cattle that are identified as FMD positive by this inspection (max, min, most likely)
- % of cattle identified as FMD positive whose test result is ignored or compromised (max, min, most likely)

____

____

Q2.36 Do you have any additional comments about antemortem inspection and detection of FMD in cattle from ${lm://Field/1} systems?

Page Break

Q2.37

Block D: Postmortem inspection and processing

${lm://Field/1} systems

Please answer the following questions considering ONLY cattle raised in ${lm://Field/1} production systems.

After each question, please use the Reasoning field to provide 1-2 sentences explaining why you chose those values.
If you do not feel comfortable answering any question, please respond "No Answer".

Q2.38 (P11) What % of carcasses undergo postmortem inspection?

- ○ Maximum: % of carcasses infected (0-100) (1) ____
- ○ Minimum (2) ____
- ○ Most likely (3) ____
- ○ Reasoning (4) ____

Q2.39 (P12) What % of FMD-infected carcasses displaying lesions are identified as FMD-positive by this inspection? (In other words, what is the sensitivity of this inspection to detect FMD in this population of carcasses?)

- ○ Maximum: % of infected carcasses detected (0-100) (1) ____
- ○ Minimum (2) ____
- ○ Most likely (3) ____
- ○ Reasoning (4) ____

Q2.40 (P13, P14) Among carcasses processed for de-boned meat, what % have lymph node and bone tissue completely removed?

○ Maximum: % of carcasses (0-100) (1) ____

○ Minimum (2) ____

○ Most likely (3) ____

○ Reasoning (4) ____

Q2.41 Is the answer to the previous question different for carcasses that were inspected vs. for those which were not inspected?

○ Yes (1) ____

○ No (2) ____

Q2.42 (P15, P16) Among carcasses processed for de-boned meat, what % undergo a maturation process which effectively destroys FMDV in muscle tissue?

○ Maximum: % of carcasses (0-100) (1) ____

○ Minimum (2) ____

○ Most likely (3) ____

○ Reasoning (4) ____

Q2.43 Is the answer to the previous question different for carcasses that were inspected vs. for those which were not inspected?

- ◯ Yes (1) ____
- ◯ No (2) ____

Q2.44
Do you have any additional comments about postmortem inspection and processing meat of cattle from ${lm://Field/1} systems?

____

End of Block: Sub-pop block
